# Review of methodologies and a protocol for the *Agrobacterium*-mediated transformation of wheat

**DOI:** 10.1186/1746-4811-1-5

**Published:** 2005-09-05

**Authors:** Huw D Jones, Angela Doherty, Huixia Wu

**Affiliations:** 1 CPI Division, Rothamsted Research, Harpenden, AL5 2JQ, UK

## Abstract

Since the first report of wheat transformation by *Agrobacterium tumefaciens *in 1997, various factors that influence T-DNA delivery and regeneration in tissue culture have been further investigated and modified. This paper reviews the current methodology literature describing *Agrobacterium *transformation of wheat and provides a complete protocol that we have developed and used to produce over one hundred transgenic lines in both spring and winter wheat varieties.

## Introduction

Transformation of cereal crops is a powerful research tool for gene discovery and function to investigate genetically controlled traits and is fast becoming a key element in the process of varietal improvement. It provides key underpinning knowledge to inform and short-cut conventional breeding strategies. For specific crops, it also enables the introduction of novel genes directly into locally-adapted germplasm and the creation of new genetically modified varieties. As testament to this, a total of 81 million Ha of approved GM crops, mainly for herbicide tolerance or insect resistance, were planted in 2004 [1], although wheat does not currently form part of this portfolio.

Wheat was among the last of the major crops to be transformed with the first fertile transgenic plants being reported using particle bombardment little over a decade ago [2-6]. Advances in the design of micro-projectile devices, choice of explant, media composition and selection systems has enabled the application of wheat transformation to study the role specific genes in a wide range of agronomically important traits (reviewed by [7-9]). Particle bombardment remains a robust, relatively efficient method for the genetic manipulation of wheat [10], however at the molecular level, the DNA integration sites are often unnecessarily complex. There are several significant advantages to transferring DNA via *Agrobacterium*, including a reduction in transgene copy number, the stable integration with fewer rearrangements of long molecules of DNA with defined ends and the ability to generate lines free from selectable marker genes [7,11-14]. This has been a driving force in the development of methods using *Agrobacterium tumefaciens *to deliver DNA although the ability to routinely transform wheat in this way is currently restricted to a few, well-resourced public and commercial laboratories worldwide. This is partly due to the need for experienced personnel and expensive laboratory and plant growth infrastructure but also through a lack of clearly-written, complete, publicly-available protocols. There are several research papers and patents describing specific improvements to methodologies but these fail to provide a step-by-step guide to the transformation process as a whole.

We have compared the published literature under headings that describe the main variables in the transformation process. First, we consider the relatively narrow range of wheat genotypes that have been successfully transformed, the choice of explant and the pre-treatments that were carried out. Second, we compare the *Agrobacterium *strains, resident Ti plasmids and binary vectors used and consider the importance of additional virulence genes. The various inoculation and co-cultivation conditions are discussed and finally the key steps to control the overgrowth of *Agrobacterium *cells and the selection of regenerating transformed plants are described. We then provide a detailed protocol for the transformation of freshly isolated immature embryos and regeneration of fertile plants in 9–12 weeks.

### Genotype and explant pre-treatments

Immature embryos of Bobwhite, pre-cultured for between 1 and 6 days on CM4C medium, are the most commonly used explant [15-18], although the use of 9 day pre-cultured immature embryos of cv. Fielder [19] and callus derived from immature embryos of Bobwhite [17] and cv. Veery 5 [20] has also been reported (see Table 1 for summary). Although immature embryos of Bobwhite are commonly pre-cultured prior to inoculation, Cheng *et al. *[17] report no significant difference in transformation efficiencies between immature embryos, pre-cultured ones or embryogenic callus. In an alternative approach, freshly isolated immature embryos of the winter and spring wheat cultivars Florida and Cadenza were found preferable to pre-cultured ones [21] and it is this explant type that is described in the accompanying protocol as it has potential to be applied to other varieties. Precocious zygotic germination is a significant problem when using immature embryo explants but can be suppressed by the addition of hormones such as dicamba, abscisic acid or high levels of 2,4-D to the culture medium. Some authors specifically state that the embryo axis was removed or damaged to prevent zygotic germination [19-21]. A marked effect of embryo size/age on T-DNA delivery and regeneration has been demonstrated, with large embryos (>2 mm) giving significantly higher transient expression levels but lower regeneration frequencies [21] than smaller ones (<1.5 mm). We emphasise the need to use embryos of 0.8–1.5 mm in the accompanying protocol.

**Table 1:** Summary of main parameters reported for *Agrobacterium*-mediated transformation of wheat.

**Wheat variety (S – spring) (W – winter)**	**Explant type**	**Embryo Axis removed **	***Agrobacterium *strain (binary vector)**	Inoculation (Co-culture) *rt – room temp	**Control of *Agrobacterium *cells**	**Plant selective agent**	**Transformation Freq. (%)**	**No of plants reported**	**Refs**
Bobwhite (S)	IE (age NS*); 1–6 d PCIE; 10–25 d EC	NS*	C58-ABI (pMON18365)	3 h, 23–25°C (2–3 d, 24–26°C)	Carbenicillin (250 mg/l)	G418	1.4–4.3	>100	[17]
Bobwhite (S)	4 d PCIE	NS*	C58-ABI (pMON30139 and others)	15–30 min, 23–25°C (2–3 d, 23–25°C)	Carbenicillin (250–500 mg/l)	Glyphosate	4.4	3354	[16]
Bobwhite (S)	1–6 d PCIE; 8–30 d EC	NS*	C58-ABI (pMON18365)	5–60 min, 23–26°C (2–3 d, 24–26°C)	Carbenicillin (250 mg/l)	G418 Paromomycin Glyphosate	4.8–19	154	[18]
Bobwhite (S)	3–6 PCIE	NS*	C58C1 (pPTN155)	45 min – 3 h, 25°C (1–3 d, 25°C)	Ticarcillium; Vancomycin Cefatoxin; (50 mg/l)	G418	0.5–1.5	13	[15]
Cadenza (S) Florida (W)	0–72 h IE	Yes	AGL1 (pAL154/156)	15 min-5 h, rt* (1–5 d, 24–25°C rt*)	Timentin (160 mg/l)	PPT (L-Phosphinothricin)	0.3–3.3	44	[21]
Fielder (S)	6–9 d PCIE	Yes	AGL0 (pBGX1)	30–60 min rt* (2–3 d, 23–24°C)	Timentin (150 mg/l)	GFP, Bialaphos	1.8	4	[19]
Veery-5 (S)	14 d EC	Yes	LBA4404 (pHK21)	15 min at rt* (1 d 27°C, 2 d 22°C)	Timentin (150 mg/l)	Glufosinate ammonium	1.2–3.9	17	[20]
Vesna (S)	IE (age NS*)	NS*	LBA4404 (pTOK233) AGL1 (pDM805)	15–30 min, (3 d, 27°C)	Cefotaxime (300 mg/l)	PPT (L-Phosphinothricin)	0.13–0.41	6	[45]
Various Chinese varieties (NS*)	EC (age NS*)	NS*	AGL1 (p^UNN-2^)	30–60 min (2 d, 28°C)	Timentin (150 mg/l)	Paromomycin	3.7–5.9	44	[46]

IE – freshly isolated immature embryos; PCIE – pre-cultured immature embryos; EC – embryogenic callus; *NS not specified.

Various explant pre-treatment steps have been evaluated in attempts to improve T-DNA delivery or tissue-culture response in particular varieties. Osmotic and desiccation treatments have been evaluated and incorporated into protocols based on particle bombardment [22-26], and have also been tested for *Agrobacterium *transformation of wheat. Air-drying pre-cultured immature embryos and embryogenic callus explants during *Agrobacterium *co-cultivation increased T-DNA-delivery and suppressed *Agrobacterium *cell growth which in turn facilitated better plant cell recovery [18]. The same authors found no such advantage when explants were desiccated prior to inoculation or when osmotic conditioning was used, however other reports indicate a beneficial effect on transformation of air-drying prior to co-culture for rice suspension cell cultures [27] and sugarcane callus [28]. Osmotic conditioning on 10% sucrose prior to *Agrobacterium *inoculation caused a marked increase of GUS transient expression in pre-cultured rice calli [29] but a plasmolysis step using 20% maltose failed to improve T-DNA delivery in 10 day pre-cultured wheat embryos [30].

### *Agrobacterium tumefaciens *strains and binary vectors

The ability of particular *Agrobacterium *strains to transform plant cells is defined by their chromosomal and plasmid genomes which between them must encode all the machinery necessary for attachment and DNA-transfer. The *Agrobacterium *strains that have been successfully used for wheat transformation are based on only two chromosomal backgrounds, LBA4404 (Ach5) and C58 but these have been used with a wide range of Ti and binary plasmids. Some strains, notably AGL0 and AGL1 have been engineered to contain the so-called hypervirulent Ti plasmid, pTiBo542 harbouring additional *vir *genes originating from the *Agrobacterium *strain A281 which in its oncogenic form possesses a broad host range and a induces large, rapidly appearing tumours [31]. The strains used in the papers reviewed (see Table 2), also contain a binary and sometimes helper plasmids, often conferring yet more copies of virulence genes. A comparison of different *Agrobacterium *strains demonstrated that AGL0, a hypervirulent strain containing a disarmed pTiBo542 plasmid [32], was better at generating wheat transformants than other strains tested [19]. The ability of the Ti plasmid pTiBo542 to confer higher transformation efficiencies was first observed in dicots [33-35] and the *vir *genes from this plasmid have been widely adopted for monocot transformation vectors (reviewed by [11]). The weakly virulent *Agrobacterium *strain LBA4404, was successful in transforming wheat only when augmented by the superbinary plasmid pHK21 which possessed extra copies of *vir *B, C and G genes from pTiBo542 but not when carrying a standard binary plasmid [20]. Further evidence of the positive effect of additional *vir *genes was provided by the demonstration that a 15 Kb fragment of pTiBo542 on a pSOUP helper plasmid [36] enhanced T-DNA delivery and the production of transgenic wheat plants, even when in a hypervirulent AGL1 background already containing pTiBo542 as a resident Ti plasmid [21,37]. Although there has been a tendency to incorporate additional *vir *genes, particularly *vir*G, into binary vectors this is not always necessary, at least for cv Bobwhite, in which a large number of transgenic lines have been reported using apparently standard *Agrobacterium *strains and binary vectors [16-18]. There is also one report [15] of transformation with a normal binary in the *Agrobacterium *strain C58C1 which the authors describe as disarmed, however it is our understanding that the C58C1 strain is actually cured of its pTiC58 plasmid [38,39]. There is currently insufficient data to define precisely which *vir *genes are necessary and where they should reside for optimal wheat transformation in different genotypes. There is also scope for further research into the effect on wheat transformation of specific *Agrobacterium *mutants that have shown beneficial effects for other plant species. For example, strains containing mutations in the *vir *gene regulator *vir*G resulting in constitutive expression of this gene and presumably the other *vir *genes it regulates, gave significant increases in efficiency of transformation in tobacco and cotton [40], *Catharanthus roseus *[41] and Norway spruce [42]. This *vir*G mutant was also combined with a high copy number plasmid to further improve transformation rates in rice and soybean [43].

**Table 2:** Summary of *Agrobacterium *strains and vectors used to investigate wheat transformation.

***Agrobacteriu*m strain (binary vector)**	**Chromosomal background**	**Ti plasmid**	**Opine classification**	**Additional *vir *genes on binary or helper plasmids**	**Binary type**	**Selectable and scorable marker on T-DNA. (Promoter shown in parentheses)**
ABI (pMON18365) [17, 18]	C58	Disarmed pTiC58	Nopaline	pMON18365, none reported	normal-binary	*npt*II (E35S) GUS (E35S)
C58C1 (pPTN155) [15]	C58	Cured/disarmed?	Nopaline	pPTN155, none reported	normal-binary	*npt*II (35S) GUS (E35S)
AGL1 (pAL154/156) [21]	C58, *RecA*	pTiBo542 ΔT-DNA	Succinamopine	pAL154, 15.2 Kb fragment from pTiBo542 [47], pAL156, none	super-binary	*bar *(Ubi1) GUS (Ubi1)
AGL0 (pBGX1 and pTO134) [19]	C58	pTiBo542 ΔT-DNA	Succinamopine	pBGX1, none reported pTO134, none reported	normal-binary	*hpt *(35S) *gfp *(Ubi1) *bar *(35S) s*gfp*S65T (35S)
AGL1 (pDM805)	C58, RecA	pTiBo542 ΔT	Succinamopine	pDM805, none reported	normal-binary	*bar *(Ubi1) GUS (Act1)
						
LBA4404 (pTOK233) [45]	Ach5	DNA Disarmed pAL4404	Octopine	pTOK233, extra copy of *vir*B, *vir*C and *vir*G from pTiBo542 47, [48]	super-binary	*hpt *(35S) GUS (35S)
LBA4404 (pHK21) [20]	*RecA *Ach5	Disarmed pAL4404	Octopine	pHK21, extra copy of *vir*B, *vir*C and *vir*G from pTiBo542 [47]	super-binary	*bar *(Ubi1) GUS (Ubi1)
AGL1 (p^UNN-2^) [46]	C58, *RecA*	pTiBo542 ΔT-DNA	Succinamopine	p^UNN-2^, none reported	normal-binary	*npt*II (Ubi1))
ABI (pMON30139 and others) [16]	C58	Disarmed pTiC58	Nopaline	pMON30139, none reported	normal-binary	*aro*A:CP4 (Act1) *aro*A:CP4 (e35S+ hsp intron)

### Inoculation and co-cultivation

The *Agrobacterium *infection process is divided into two stages: first, a short period, typically a few minutes to a few hours (see Table 1), of inoculation by complete or partial immersion of explants in an *Agrobacterium *suspension. Then, after the majority of *Agrobacterium *cells are removed by pouring or pipetting, the explants are co-cultivated for a further 1–3 days. One or both these steps are carried out in darkness at approximately 25°C, although a two temperature co-cultivation step has also been tried with one day at 27°C then two days to 25°C [20]. During the co-cultivation period, phenolic inducers such as acetosyringone work alongside other signalling factors such as temperature and an acid environment to promote the expression of *vir *genes. The presence of 200 μM acetosyringone in the *Agrobacterium *or co-cultivation medium markedly increased T-DNA delivery [21]. Enhanced transient GFP expression was observed in wheat cell clusters with acetosyrigone at 400 μM in the co-cultivation but not the inoculation media [19]. The need for acetosyringone been reported for a variety of wheat explants types [17,37,44] but not for wheat cell suspension cultures where exogenous induction agents were not necessary for stable transformation [17].

The use of surfactants during inoculation and co-cultivation significantly increases T-DNA delivery. Increasing concentrations of Silwet L-77 up to 0.04% had positive effects on T-DNA delivery as measured by the number of immature embryos with GUS foci and the number of GUS foci per embryo [21]. However, concentrations higher than 0.05% reduced survival and callus formation in freshly isolated immature embryos and an optimal concentration of 0.01% was chosen [21]. Positive effects of surfactants were also reported in study [17] which used Silwet and pluronic acid F68 at 0.02%. Silwet has been used at concentrations as high as 0.05% for pre-cultured embryos and calli [15]. The protocol presented here uses Silwet L-77 at 0.015% but no pre-culture or special inoculation treatments.

### Control of *Agrobacterium*, regeneration and selection

After the co-cultivation period, infected explants progress in a series of tissue culture steps on media designed to inhibit the growth of *Agrobacterium *cells and promote regeneration and selection of transformants. The antibiotics used to control the growth of *Agrobacterium *are added immediately after co-cultivation during the callus induction phase and are maintained in all subsequent media. Timentin or carbenicillin are commonly used but other compounds such as cefatoxin, cefotaxime, ticarcillium and vancomycin have also been reported (see Table 1). Plant selection agents complementary to the marker gene on the T-DNA are introduced to kill or compromise the growth of untransformed material. Selection for plant transformation is often initiated a few days after co-cultivation during the callus-induction phase and maintained during the latter regeneration steps. Delayed selection, started at the later plant regeneration phase was preferred by [21] and is the method described in the accompanying protocol. Three selectable marker gene systems have been reported for *Agrobacterium *transformation of wheat. The first is based on antibiotic selection using either *hpt *(*aph4*-Ib) or *npt*II (*aph3*'II) which encode phosphotransferase enzymes that confer tolerance to the aminoglycoside antibiotics such as kanamycin, neomycin, paromomycin, G418 and hygromycin. A second system utilizes the bar gene which confers tolerance to glufosinate ammonium-based herbicides such as PPT, Basta, Bialaphos etc. A third system is based on the *aro*A:CP4 genes conferring tolerance to glyphosate-based herbicides such as Roundup. The use of 0.02 mM glyphosate on regenerating meristems has been reported to reduce the number of plants escaping selection to zero [16]. *Npt*II, *bar *and *aro*A:CP4 have been successfully used by different groups to produce transgenic wheat plants but it is not possible to draw direct comparisons between selection systems because often a visual marker was also used in conjunction with chemical selection. For example, in several studies, the GUS reporter gene has been used in addition to the conventional selectable marker to help optimise the identification of transformants [15,17,18,20,21,45]. Also, a T-DNA containing both *hpt *and GFP, along with hygromycin selection, has been used to identify early events in the transformation process [19].

In wheat transformation via *Agrobacterium*, the total length of time, from isolation of the original explant to the transfer of young plants to soil, is typically 12–16 weeks depending on the length of pre-culture and the number of selection steps. A shortened protocol taking only 7–11 weeks, achieved mainly by reducing the selection step to one week, has also been reported [16]. The protocol described in the present paper was optimised for *bar*/glyphosate selection with a GUS assay to confirm T-DNA integration and expression and takes approximately 12 weeks.

### Concluding remarks

The advantages arising from simple molecular integrations of single copy DNA fragments with defined ends have driven research into *Agrobacterium*-mediated plant transformation. Compared to rice and maize, progress with wheat has been slower but as described here, robust methods for the transformation of wheat using *Agrobacterium *now exist. There is scope to further optimise the media components and pH and to investigate the ideal virulence gene complement. Current bottlenecks limiting throughput include the labour-intensive steps of embryo isolation and transfers between media. Unlike biolistics, *Agrobacterium *suspensions can be manipulated by liquid handing robots and this combined with the use of callus cultures and the automation of transfer steps would enable a higher throughput which even at low efficiency would allow significantly more transgenic lines to be produced

### A protocol for wheat (*Triticum aestivum *L.) transformation mediated by *Agrobacterium tumefaciens*

#### Scope and limitations

This method was developed for the winter wheat cultivar Florida but with minor modifications has also been used to successfully transform the spring wheat varieties Fielder and Cadenza. It utilises the super-virulent *Agrobacterium tumefaciens *strain AGL1 [32] containing the plasmids pAL154/pAL156 which are based on the plasmid pSoup/pGreen [36], . The binary vector pAL156 contains a single T-DNA incorporating the *bar *gene conferring Basta resistance and a modified *uid*A (GUS) gene with an intron within the open reading frame to prevent its expression in *Agrobacteium *itself. Both the *bar *and *uid*A genes are driven by the maize ubiquitin1 promoter plus ubiquitin1 intron [49]. The *bar *gene is located next to the left border, and *uid*A is adjacent to the right border. A helper plasmid pAL154 provides replication functions for pAL156 *in trans *and also contains the 15 kb Komari fragment [35,47] supplying extra *vir *genes. Other *Agrobacteium *strains and plasmid combinations may also be appropriate in our protocol but have not yet been tested.

**Figure 1:**
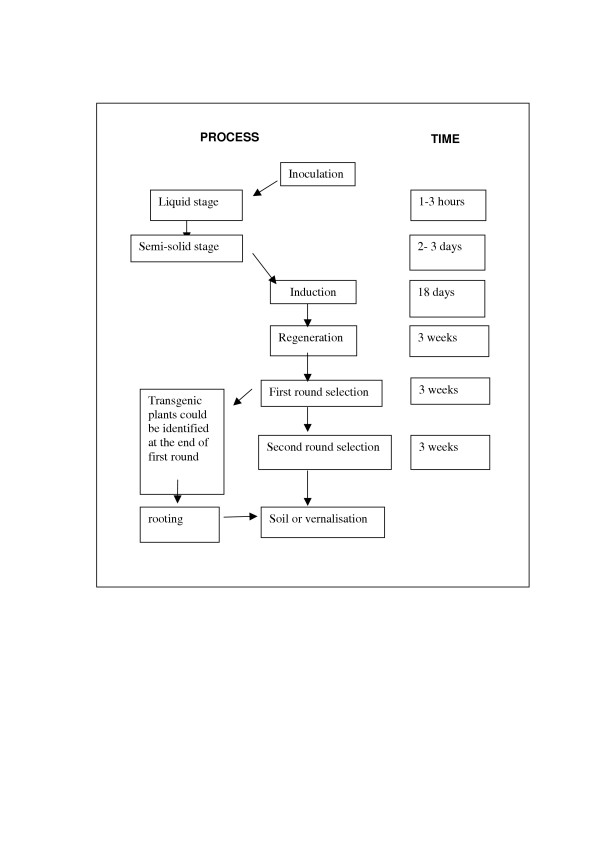
Main steps in the protocol for *Agrobacterium *transformation of wheat from inoculation to the transfer of transgenic wheat plants to soil.

There are three main steps in the method: 1. incubation of freshly-isolated immature embryos with *Agrobacterium tumefaciens*; 2. induction of embryogenic callus and regeneration of shoots and roots; 3. application of a herbicide selection system to allow only the transgenic plantlets to survive. The average efficiency of transformation (number of independent transgenic lines/total number of immature embryos inoculated) is approximately 1%. The protocol takes 9–12 weeks from the isolation of immature embryos to the potting of putative transgenic plantlets to soil (Figure 1).

### Protocol

#### Growth of donor plants

1.1 Sow seeds, 4–5 per 21 cm diameter pot, in compost which contains 75% fine-grade peat, 12% screened sterilised loam, 10% 6 mm screened lime-free grit, 3% medium vermiculite, 2 kg Osmocote Plus/m3 (slow-release fertiliser, 15N/11P/13K plus micronutrients), 0.5 kg PG mix/m3 (14N/16P/18K granular fertiliser plus micronutrients (Petersfield Products, Leicestershire, UK). Although other soil formulations may also be suitable.

1.2 Grow wheat plants in environmentally controlled growth rooms for approximately 11 weeks to provide immature seeds.

1.3 Growth rooms are maintained at 18–20°C day and 14–15°C night temperatures with a relative air humidity of 50–70% under a 16 h photo-period provided by banks of 400 W High Temperature Quartz Iodine lamps (Osram Ltd., Berkshire, UK) which give light intensity ~700 μmolm^-2^s^-1 ^photosynthetically active radiation (PAR).

1.4 Before transferring to these conditions, winter wheat varieties are vernalised from seed for 8 weeks at 4–5°C with a 12 hour photoperiod provided by 70 W fluorescent lamps giving approximately 150 μmolm^-2^s^-1 ^PAR at 300 mm from the lights.

1.5 The water is supplied by an automated flooding system, but seedling-stage plants are initially top watered individually for a few weeks [50].

#### 2 Growth and preparation of Agrobacterium cells for inoculation

2.1 Initiate *Agrobacterium *liquid cultures by adding ~200 μl of a standard glycerol inoculum to 10 ml MG/L [51] (Table 3) plus antibiotics. Prepare as many 10 ml cultures as plates to be treated.

**Table 3:** Composition of medium MG/L

**Component**	**/litre**
Mannitol	5 g
L-Glutamic acid	1 g
KH_2_PO4	250 mg
NaCl	100 mg
MgSO_4_·7H_2_O	100 mg
Tryptone	5 g
Yeast extract	2.5 g
pH 7.0	
Biotin (added after autoclaving from stock at 1 mg/100 ml (add 100 μl to 1 litre MG/L)	1 μg

2.2 Incubate at 27–29°C, shaking (250 rpm) for 12–24 hours (to reach an OD >1 (Abs = 600 nm)).

2.3 Pellet the *Agrobacterium *culture at 4500 g for 10 minutes and resuspend in 4 ml single-strength inoculation medium (see 6.2.2) supplemented with 200 μM acetosyringone for each 10 ml culture.

2.4 Replace the cultures back on the shaker until required, but they should be used within 3 hours.

Note, The antibiotics used depend on the selectable markers in the *Agrobacterium *strain and binary vectors used. For the AGL1 strain used in this protocol, carbenicillin (200 mg/l) is used and pAL154/156 combinations are selected with kanamycin (100 mg/l) which is the selectable marker on pAL156.

#### 3 Preparation of explants

##### 3.1 Ear collection and surface sterilization

3.1.1 Collect ears at approximately 12–16 days post-anthesis, a few seeds can be opened at the time of collection to determine the size and texture of the embryos, which should be 0.8 – 1.5 mm in length and translucent in appearance.

3.1.2 Surface sterilise by rinsing in 70% (v/v) aqueous ethanol for 1 minute then 15 minutes in 10% (v/v) Domestos bleach solution (Lever) with gentle shaking. Rinse with sterile distilled water at least three times.

Note, due to asynchronous development, only half or two thirds of the seeds on any one ear will be suitable, the seeds nearest to the peduncle are generally younger and smaller.

##### 3.2 Isolation of immature embryos

3.2.1 Isolate the embryos from the seed under a stereo microscope in a sterile environment using a sharp scalpel.

3.2.2 Remove and discard the embryo axis first then isolate the remaining portion of the embryo which is now referred to as the scutellum.

3.2.3 Plate scutella with the axis side (now removed) down onto semi-solid inoculation medium in 55 mm Petri dishes, about 50 scutella per plate.

3.2.4 It is important to inoculate each plate of 50 scutella with *Agrobacterium tumefaciens*, as described below, before isolating embryos for the next plate.

#### 4 Inoculation of scutella with *Agrobacterium tumefaciens*

4.1 Take the resuspended *Agrobacterium *suspension from the shaker, add 60 μl 1% Silwet to make a final concentration of 0.015% and pour the whole 4 ml over a batch of 50 plated scutella.

4.2 Incubate for 1–3 hours at room temperature while preparing more scutella for inoculation as described in 3.2.

4.3 Transfer the scutella without blotting, keeping the ex-axis side down, onto fresh inoculation medium in 55 mm dishes. Allow to co-cultivate in the dark at 22–23°C for 2–3 days.

#### 5 Control of *Agrobacterium *and induction of embryogenic calli, regeneration and selection

5.1 After 2–3 days co-cultivation, transfer all scutella to induction medium (Table 4) and continue to incubate in the dark at 24–25°C.

**Table 4:** Composition of double-strength culture media. All concentrations are shown double-strength except for the supplements added after pH adjustment and sterilisation which are shown at their final concentrations.

**Component**	**Inoculation (/L)**	**Induction (/L)**	**RDZ (/L)**	**RPPT (/L)**	**R (/L)**
MS Macro salts (×10)	200 ml	200 ml	200 ml	200 ml	200 ml
L7 Micro salts (×1000)	2 ml	2 ml	2 ml	2 ml	2 ml
FeNaEDTA (×100)	20 ml	20 ml	20 ml	20 ml	20 ml
MS vitamins (×1000)	2 ml	2 ml	-	-	-
Vitamins/Inositol (×200)	-	-	10 ml	10 ml	10 ml
Inositol	200 mg	200 mg	200 mg	200 mg	200 mg
Glutamine	1 g	1 g	-	-	-
Casein hydrolysate	200 mg	200 mg	-	-	-
MES	3.9 g	3.9 g	-	-	-
Glucose	20 g	-	-	-	-
Maltose	80 g	80 g	60 g	60 g	60 g
	pH adjusted to 5.8 then autoclaved		pH adjusted to 5.7 then filter sterilised		
2,4-D	2 mg	0.5 mg	0.1 mg	-	-
Picloram	2.0 mg	2.0 mg	-	-	-
Acetosyringone	200 μM	-	-	-	-
Timentin	-	160 mg	160 mg	160 mg	160 mg
Zeatin	-	-	5 mg	-	-
PPT	-	-	-	2–4 mg	3–4 mg

5.2 After 18 days, transfer embryogenic calli to RDZ medium (Table 4), and incubate at 24–25°C but in the light. Embryogenic calli derived from the same immature embryo should be kept intact without breaking up.

5.3 After 3 weeks, transfer embryogenic calli to selection medium RPPT (or appropriate selection agent, Table 4). At this point, the calli can be broken into defined shoots/roots, but it is important to keep these together, or mark them clearly as there is possibility that these may be clones.

5.4 Continue transferring to fresh RPPT every 3 weeks until PPT tolerant plantlets are ready to be potted to soil.

Note, at the end of the first round of selection, some of the transgenic plants may be identified by GUS assay on leaf fragments. If they have good strong roots, they may be transferred to soil or put into the vernalisation room immediately, otherwise, transfer them to R medium without PPT for root strengthening (Table 4).

#### 6 Materials

##### 6.1 Media for growing Agrobacterium tumefaciens

See Table 3.

##### 6.2 Media for plant tissue culture

6.2.1 Plant tissue culture media are prepared from stock solutions at double strength to allow the addition of an equal volume of gelling agent; Phytagel for inoculation and induction media, agargel for RDZ, RPPT, and R media. Gelling agents are also prepared at double strength (Phytagel at 4 g/l and agargel at 10 g/l) and autoclaved at 121°C for 20 min (see Table 4).

6.2.2 To make single-strength liquid inoculation media for resuspending *Agrobacterium *cells in section 2.3, simply mix double-strength medium with autoclaved, distilled water.

##### Stock solutions for basal culture media

Detailed below are the recipes for stock solutions of basal culture media components adapted from [50].

6.2.3 MS Macrosalts (×10):

16.5 g/l NH_4_NO_3 _(Fisher Scientific, Leicestershire, UK),

19.0 g/l KNO_3 _(Sigma-Aldrich, Dorset, UK),

1.7 g/l KH_2_PO_4 _(Fisher Scientific UK),

3.7 g/l MgSO_4_·7H_2_O (Fisher Scientific UK),

4.4 g/l CaCl_2_·2H_2_O (Fisher Scientific UK).

Note, Dissolve each component in distilled water separately before mixing. Autoclave at 121°C for 20 min and store at 4°C.

6.2.4 L7 Microsalts (×1000):

15.0 g/l MnSO_4 _(Fisher Scientific UK),

5.0 g/l H_3_BO_3 _(Fisher Scientific UK),

7.5 g/l ZnSO_4_·7H_2_O (Fisher Scientific UK),

0.75 g/l KI (Fisher Scientific UK),

0.25 g/l Na_2_MoO_4_·2H_2_O (VWR International Ltd., Leicestershire, UK),

0.025 g/l CuSO_4_·5H_2_O (Fisher Scientific, UK),

0.025 g/l CoCl_2_·6H_2_O (Sigma-Aldrich).

Note, MnSO_4 _may have various hydrated states which will alter the required weight. For MnSO_4_·H_2_O, add 17.05 g/l, for MnSO_4_·4H_2_O, add 23.22 g/l, for MnSO_4_·7H_2_O, add 27.95 g/l. Prepare 100 ml microsalt stock solution at a time. Filter sterilise, and store at 4°C.

6.2.5 MS Vitamins (-Glycine) (×1000):

0.1 g/l Thiamine HCl (Sigma-Aldrich),

0.5 g/l Pyridoxine HCl (Sigma-Aldrich),

0.5 g/l Nicotinic acid (Sigma-Aldrich).

Prepare 100 ml at a time. Filter sterilise, and store at 4°C.

6.2.6 Vitamins/Inositol (×200):

40.0 g/l Myo-Inositol (Sigma-Aldrich),

2.0 g/l Thiamine HCl (Sigma-Aldrich),

0.2 g/l Pyridoxine HCl (Sigma-Aldrich),

0.2 g/l Nicotinic acid (Sigma-Aldrich),

0.2 g/l Ca-Pantothenate (Sigma-Aldrich),

0.2 g/l Ascorbic acid (Sigma-Aldrich).

Filter sterilize and store at -20°C in 10 ml aliquots.

6.2.7 Supplements

• Acetosyringone (3',5'-Dimethoxy-4'-hydroxyacetophenone) (Aldrich D12,440-6: MW-196.20), Dissolve in 70% ethanol to give 10 mg/ml or 50 mM stock solution. Filter sterilise, aliquot and store at -20°C.

• 2,4-Dichlorophenoxyacetic acid (2,4-D) (Sigma-Aldrich), 1 mg/ml in ethanol/water (dissolve powder in ethanol then add water to volume). Filter sterilise, and store at -20°C in 1 ml aliquots.

• Zeatin mixed isomers (10 mg/ml) (Sigma-Aldrich), Dissolve powder in small volume 1 M HCl and make up to volume with water, mix well/vortex. Filter sterilise, and store at -20°C in 1 ml aliquots.

• Picloram (1 mg/ml) (Sigma-Aidrich), Dissolve picloram in water, filter sterilise and store at -20°C in 2 ml aliquots.

• Timentin (300 mg/ml) (Melford, UK), Dissolve Timentin (Ticarcillin/Clavulanic (15:1)) in water, filter sterilise and store at -20°C in 1 ml aliquots.

• PPT (10 mg/ml)(Glufosinate Ammonium) (Melford, UK), Dissolve in water, mix well/vortex, filter sterilize, and store at -20°C in 1 ml aliquots.

• Silwet L-77 (1% v/v) (Lehle seeds, USA), Dissolve in water, filter sterilize, and store at 4°C in 0.5 ml aliquots.

## Competing interests

The author(s) declare that they have no competing interests.
